# Methyltransferase‐like 3 mediated RNA m^6^A modifications in the reproductive system: Potentials for diagnosis and therapy

**DOI:** 10.1111/jcmm.18128

**Published:** 2024-02-08

**Authors:** Xiaojuan Su, Ruifeng Lu, Yi Qu, Dezhi Mu

**Affiliations:** ^1^ Department of Pediatrics/Key Laboratory of Birth Defects and Related Diseases of Women and Children (Ministry of Education) West China Second University Hospital, Sichuan University Chengdu China; ^2^ NHC Key Laboratory of Chronobiology (Sichuan University) Chengdu China

**Keywords:** assisted reproduction failure, asthenozoospermia, cervical cancer, endometrial cancer, follicle development, gametogenesis, germ cell tumour, methyltransferase‐like 3, ovarian carcinoma, spermatogenesis

## Abstract

Several studies have highlighted the functional indispensability of methyltransferase‐like 3 (METTL3) in the reproductive system. However, a review that comprehensively interprets these studies and elucidates their relationships is lacking. Therefore, the present work aimed to review studies that have investigated the functions of METTL3 in the reproductive system (including spermatogenesis, follicle development, gametogenesis, reproductive cancer, asthenozoospermia and assisted reproduction failure). This review suggests that METTL3 functions not only essential for normal development, but also detrimental in the occurrence of disorders. In addition, promising applications of METTL3 as a diagnostic or prognostic biomarker and therapeutic target for reproductive disorders have been proposed. Collectively, this review provides comprehensive interpretations, novel insights, potential applications and future perspectives on the role of METTL3 in regulating the reproductive system, which may be a valuable reference for researchers and clinicians.

## 1 BACKGROUND

The reproductive system is primarily responsible for offspring production and ensures gene flow from one generation to the next.^1^ It relies on the intricate performance of male and female reproductive organs, including spermatogenesis, follicle development, gametogenesis, embryonic formation and development.^1^ In mammals, germline development relies on the precise regulation of gene expression at the transcriptional, posttranscriptional and translational levels to ensure the formation of normal haploid gametes.^2^ At present, mainly due to abnormal gamete development and tumorigenesis in the reproductive organs, infertility or phenotypic differences often arise despite the transference of genetic information from both parents to their offspring.^3^ However, the mechanisms underlying these intricate regulatory processes remain unclear.

Differences in gene expression or phenotype occurrence may be attributed to epigenetic modifications, such as the prevalent N^6^‐methyladenosine (m^6^A) modification of RNA molecules, which has received increasing attention in the investigation of the mechanism of biological processes.^4^, ^5^ RNA m^6^A is one of the most prominent epigenetic modification, and is associated with three regulatory factors: writers, erasers and readers.^4^, ^5^ Writers are commonly known for the activity of methyltransferases to add methyl groups (*–CH*_*3*_), whereas erasers are primarily demethylases with the opposite ability, to remove *–CH*_*3*_, and readers primarily recognize m^6^A RNA and decide the fate of RNA.^4^, ^5^ Writers, also called m^6^A methyltransferase complex, consists of a multicomponent that is primarily installed by methyltransferase‐like 3 (METTL3) and methyltransferase‐like 14 (METTL14), as well as some other regulatory subunits such as the Wilms' tumour 1 (WT1) associated protein (WTAP).^6^ As the core components of the methyltransferase complex, METTL3 and METTL14 form a stable heterodimer complex.^6^ METTL3 is the sole catalytic subunit that utilizes S‐adenosylmethionine as the methyl donor; whereas METTL14 stabilizes METTL3 and plays a structural role in RNA recognition by readers, which independent of the RNA m^6^A methyltransferase activity.^7^ WTAP functions to regulate the localization to the nuclear speckle and recruitment of RNAs for the methyltransferase complex.^6^ The erasers control the nucleus exportation and metabolism of RNAs.^8^ Besides, the methylated RNAs are selectively recognized by readers, primarily the N^6^‐methyladenosine RNA binding proteins (YTHDF) and YTH domain‐containing proteins (YTHDC).^9^ Therefore, m^6^A modification is a crucial regulator that responsible for RNA biological processes, affecting various physiological processes and disease progression.^10^

**FIGURE 1:**
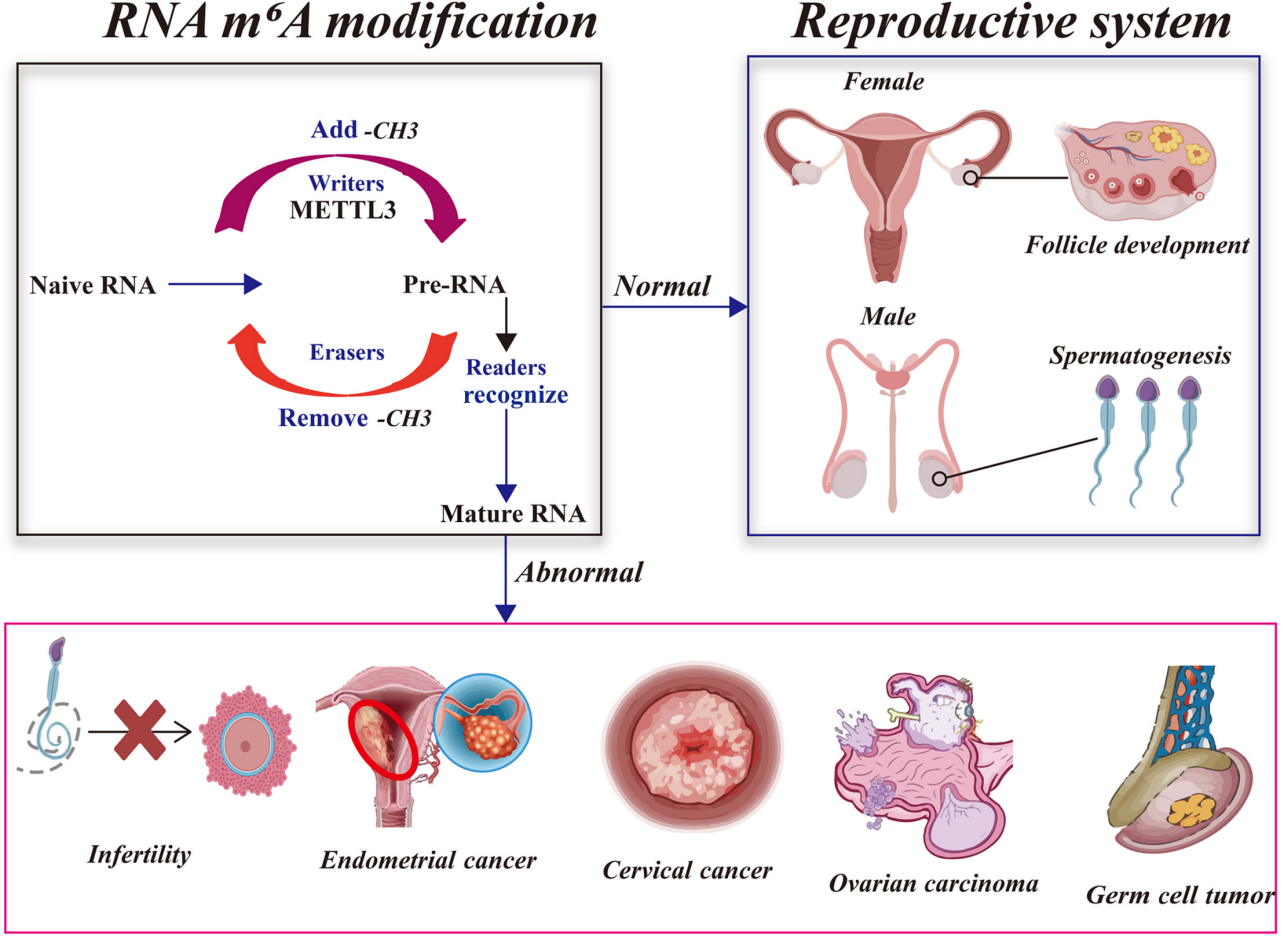
Role and mechanism of METTL3‐mediated RNA m^6^A modification in the reproductive system. The mechanism of RNA m^6^A modification is regulated by writers (functions to add –*CH*_*3*_), erasers (functions to remove –*CH*_*3*_), and readers (functions to recognize the target RNA), which processing RNAs from naive to the mature state. METTL3 is the core component of the m^6^A writers that participate in various physiological processes and disease progression. The biological functions, mechanisms and clinical potential of METTL3‐mediated RNA m^6^A modification in reproductive system, including normal (spermatogenesis, follicle development and gametogenesis) and abnormal events (cancer, infertility and assisted reproductive failure), are interpreted. –*CH*_*3*_, methyl group; m^6^A, N^6^‐methyladenosine; METTL3, methyltransferase‐like 3.

Several studies have reported the implication of METTL3 in the reproductive system, including physiological processes, such as spermatogenesis, follicle development and gametogenesis, as well as pathological processes, such as asthenospermia, assisted reproductive failure and cancer (cervical cancer [CC], endometrial cancer [EC],^17^ ovarian carcinoma [OVC]^18^ and germ cell tumour [GCT]).^11^, ^12^, ^13^, ^14^, ^15^, ^16^, ^17^, ^18^, ^19^ However, a comprehensive review summarizing and interpreting the roles of METTL3 in the regulation of the reproductive system is still lacking. A comprehensive review is usually helpful and convenient for researchers by providing them with systematic and cutting‐edge information. The current review therefore aimed to provide this by reviewing studies that have investigated the function of METTL3 in the reproductive system. Importantly, a comprehensive interpretation of these studies was performed, and the regulatory network of METTL3 in the reproductive system was mapped to collectively elucidate the relationship (Figure 1).

## 2 METTL3 MAINTAINS THE NORMAL FUNCTIONS OF THE REPRODUCTIVE SYSTEM

### 2.1 METTL3 is required for spermatogenesis

**FIGURE 2:**
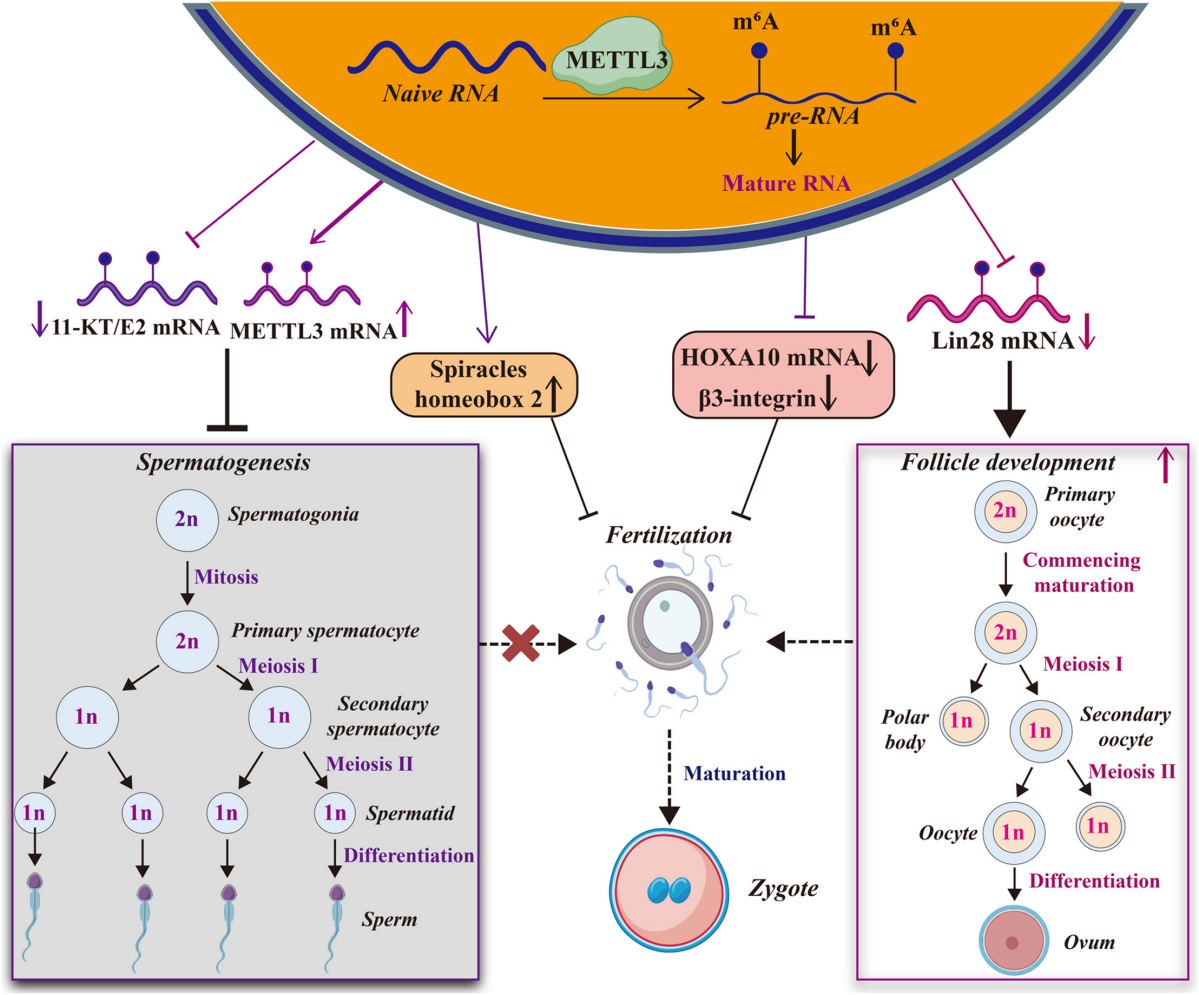
METTL3 maintains the normal functions of human reproduction. METTL3 processes RNAs from the naive state into the mature state by mediating m^6^A modification, regulating RNA biological functions such as the decay, stability and translation. Subsequently, these methylated RNAs participate in the different events of human reproduction, including spermatogenesis, follicular development, and gametogenesis. 11‐KT/E2, 11‐ketotestosterone/17b‐estradiol; HOXA10, homeobox A10; METTL3, methyltransferase‐like 3.

Male gametes, also known as spermatozoa, are endowed with a flagellum, an elongated, motile tail‐like projection.^20^ METTL3 plays a pivotal role in spermatogenesis by regulating the differentiation of spermatogonia and the initiation of meiosis. Xu et al.^21^ demonstrated that depletion of METTL3 in germ cells impairs normal spermatogonial differentiation and disrupts meiotic initiation. Mechanistically, deletion of METTL3 has been shown to have a profound impact on gene expression and alternative splicing during spermatogenesis.^21^ Moreover, Rowe et al.^22^ reported that absence of METTL3 leads to the mislocalization of *Drosophila* spermatids and aberrations in spermatogenesis. Collectively, these studies offer new insights into the roles and mechanisms underlying METTL3‐mediated m^6^A modifications in spermatogenesis and reproduction (Figure 2).

### 2.2 METTL3 contributes to follicle development

Female gametes, also known as ova or eggs, are immotile and comparatively larger than spermatozoa.^23^ In addition to its role in regulating spermatogenesis, METTL3 plays crucial roles in oocyte division, differentiation, maturation and follicle development. Notably, based on a study conducted on pig oocyte development, during meiotic maturation (from the germinal vesicle to metaphase II [MII] stages), the transcript levels of METTL3 were found to be elevated and predominantly located in the ooplasm.^24^ Mechanistically, the chemically induced reduction of m^6^A methylation in nucleic acids during porcine oocyte meiosis may hinder meiotic maturation and subsequent developmental potential.^24^ This could be attributed to a decrease in the abundance of the pluripotency marker *Lin28* mRNA and the disruption of MPF‐regulated chromosome/spindle organization.^24^

With the advancement of sequencing, typically the single‐cell methylated RNA immunoprecipitation sequencing (MeRIP‐seq/m^6^A‐IP), the capture of the transcriptome‐wide m^6^A landscape and comparison of the m^6^A level among single cells is possible. Yao et al.^25^ found that oocyte‐specific deletion of METTL3 causes m^6^A downregulation and abnormal RNA clearance, which consequently induces the formation of oocytes with low quality. In detail, the METTL3‐mediated m^6^A modification regulates the translation and stability of mRNAs (such as *Bnc2, Tmed9, Arcn1 and Cd320*) in MII oocytes by binding to YTHDF3 and regulates oocyte‐to‐embryo transition by binding to insulin like growth factor 2 mRNA binding protein 2 (IGF2BP2), which finally hinders the maturation of oocyte.^25^ In line with this study, Wu et al.^12^ revealed that deletion of METTL3, specifically in oocytes, resulted in DNA damage and accumulation, deficiency in follicle development and abnormal ovulation.^12^ Mechanistically, METTL3‐mediated m^6^A modification enhanced intersectin 2 (*Itsn2*) stability and influenced oocyte meiosis.^12^ Collectively, METTL3 functions to methylate mRNAs (*Bnc2, Tmed9, Arcn1, Cd320 and* Itsn2) by interacting with the reader proteins, which exerted recognition on these mRNAs. These findings highlight that METTL3 regulates follicle maturation and development by targeting *Bnc2, Tmed9, Arcn1, Cd320 and* Itsn2 during oocyte growth. Furthermore, Sui et al.^26^ reported that downregulation of METTL3 in female germ cells hinders oocyte maturation by inhibiting mRNA translation and impairs maternal‐to‐zygotic transition by disrupting mRNA degradation. These findings suggest that the reversible m^6^A modification of mRNA plays a pivotal role in mammalian oocyte maturation and embryonic development before implantation. To summarize, METTL3 plays pivotal role in ensuring proper oocyte development, as its absence hinders the meiosis and maturation process of oocyte (Figure 2).

### 2.3 METTL3 is indispensable for normal gametogenesis

Gametes, the reproductive or sex cells that merge during sexual reproduction to create a novel cell known as a zygote via meiosis, are produced through an intricately regulated and highly complex process of mitosis, meiosis and gametogenesis in mammals.^27^

Xia et al.^28^ reported that oocytes in adult females with *METTL3* mutation were arrested at an early stage of development, and the proportion of fully grown follicles was remarkably lower than those in individuals without a *METTL3* mutation. Human chorionic gonadotropin‐induced germinal vesicle breaks down in vitro, and the number of eggs ovulated in vivo decreases, whereas defects in oocyte maturation can be repaired by sex hormones both in vitro and in vivo.^28^ In adult males with *METTL3* mutations, sperm maturation is consistently impaired, and sperm motility is remarkably hindered.^28^ This defect can be attributed to decreased levels of 11‐ketotestosterone and 17b‐estradiol (11‐KT/E2), dysregulated expression of *11‐KT/E2* mRNA that are crucial for sex hormone generation, and the disrupted gonadotropin signalling, which are accompanied by a decrease in the overall m^6^A modification levels.^28^ Besides, embryonic stem cells (ESCs) with *METTL3* mutation extensively enhance the synthesis of nascent transcripts and result in the early mouse embryonic lethality.^29^ MeRIP‐seq results found that the whole level of m^6^A is reduced and the chromosome‐associated regulatory mRNAs (*Prdm9, Kmt2d, Esrrb and Ranbp17*) are increased after *METTL3* mutation.^29^ Mechanistically, YTHDC1 recognizes these m^6^A‐methylated mRNAs that negatively binds to EP300 and YY1 and triggers their decay through the nuclear exosome targeting complex in ESCs, finally regulates the chromatin state and transcription to influence the differentiation of ESCs.^29^ Furthermore, by analysing the dense SNP genotyping data, Flori et al.^30^ demonstrated that *METTL3* variants are a candidate gene that contributes to the variations of phenotype in cattle offspring. These findings provide evidence that *METTL3* mutations result in unsuccessful gamete maturation, embryonic lethality, fertility deficiency, as well as variations in offspring, indicating that METTL3 and m^6^A modifications are indispensable for optimal reproduction in vertebrates (Figure 2).

In conclusion, upon thorough review of these primary publications, it was inferred that upholding a relatively elevated level of METTL3 expression would prove advantageous in fostering the differentiation and maturation of spermatogonia, oocytes, and gametes, which are all crucial components for normal spermatogenesis, follicular development, and gametogenesis.

## 3 METTL3 INDUCES ASTHENOZOOSPERMIA AND REPRODUCTION FAILURE

### 3.1 METTL3 triggers asthenozoospermia occurrence

Asthenozoospermia is a deficiency in sperm quality, resulting from a reduction in the proportion of motile spermatozoa commonly referred to as sluggish or indolent sperm.^31^ Occasionally, this anomaly co‐occurs with oligospermia (low concentration of spermatozoa in ejaculated semen), known as oligoasthenozoospermia.^32^ One study revealed that the expression of *METTL3* mRNA in patients with asthenozoospermia was significantly higher than that in controls, which impacts sperm motility and constitutes a detrimental role for asthenozoospermia.^33^

Furthermore, a separate study revealed that melatonin plays a crucial role in rescuing spermatogonial stem cell mitophagy induced by Cr (VI) with the assistance of METTL3‐mediated RNA m^6^A modification.^34^ Mechanistically, METTL3 activated the mitochondrial fusion proteins (i.e. MFN2 and OPA1) while simultaneously suppressing the BCL2 interacting protein 3 (BNIP3)/NIX receptor pathway that responsible for mitophagy.^34^ Therefore, these findings offer novel insights into the molecular mechanisms for METTL3‐mediated RNA m^6^A modification in gene expression regulation that maintains the mitochondrial dynamic balance during Cr (VI)‐induced male fertility damage (Figure 2).

### 3.2 METTL3 is a risk factor for recurrent implantation failure

Furthermore, another study demonstrated that METTL3 expression is elevated in the endometrial tissues of women that experiencing recurrent implantation failure (RIF) when compared with that in controls.^35^ This elevation reduces the ratio of BeWo spheroid attachment. Mechanistically, METTL3 catalyses the m^6^A methylation of homeobox A10 (*HOXA10*) mRNA and suppresses HOXA10 expression, while increasing the expression of empty spiracle homeobox 2 and decreasing the level of β3‐integrin.^35^ These findings suggest that the upregulation of METTL3 has a detrimental impact on embryo implantation by inhibiting HOXA10 expression, thereby contributing to RIF pathogenesis. Besides, decidualization is a key step for establishing receptivity that provides appropriate conditions for embryo implantation, abnormal of which usually causes RIF or infertility.^36^, ^37^ Study found that the abnormal expression of METTL3 leads to the degradation of *FOXO1* mRNA through binding to YTHDF2, which subsequently affecting cellular decidualization and embryo implantation^38^ (Figure 2). Collectively, the upregulated expression of METTL3 triggers the occurrence and progression of asthenozoospermia, RIF, and infertility, indicating that a high level of METTL3 may serve as a risk indicator for these diseases as well as a potential diagnostic biomarker and therapeutic target in clinical settings.

## 4 METTL3 MIGHT BE A POTENTIAL ONCOGENE IN REPRODUCTIVE TUMOURS

### 4.1 Oncogenic role of METTL3 in CC: A biomarker for diagnosis and prognosis as well as a target for therapy

CC is the most prevalent gynaecological malignant tumour and has three common types: Squamous cell carcinoma, adenocarcinoma and adenosquamous carcinoma.^39^ CC arises from persistent infection with certain strains of the human papillomavirus (HPV), such as HPV16 and HPV18.^40^ Recent studies have revealed that patients with a high expression of METTL3 and cluster of differentiation 33^+^ (CD33^+^) myeloid‐derived suppressor cells (MDSCs) in their CC tumour tissues exhibit a marked contrast to that in adjacent tissues.^41^

Importantly, elevated levels of METTL3 within tumour microenvironments significantly correlated with the advanced tumour stage, poor overall survival (OS), and poor disease‐free survival (DFS) in patients with CC.^41^ This is an independent prognostic factor for patient survival, particularly DFS and OS, whereas the CD33^+^ MDSCs count serves as an autonomous predictor of DFS.^41^ Collectively, these findings suggest that the expansion of CD33^+^ MDSCs is associated with elevated levels of METTL3 and both factors serve as independent prognostic indicators in CC. In line with this study, Yu et al.^16^ conducted an integrative analysis of m^6^A regulators in CC and found that METTL3 was overexpressed in CC and positively linked to tumour HPV status (i.e., HPV integration status, E6 and unspliced‐E6 expression, p16 expression). Mechanistically, the enhanced level of METTL3 inhibited tumour immune cell infiltrations and upregulated PD‐L1 expression.^16^ Collectively, these findings suggest that METTL3 controls the immunosuppressive tumour microenvironment in HPV‐induced CC, highlighting METTL3 as a potential therapeutic target for anti‐cancer immunotherapy. Furthermore, Wu et al.^42^ reported that increased METTL3 expression serves as an independent predictor of unfavourable outcomes among early‐stage squamous cells in patients with CC. These studies collectively proposed METTL3 as a promising diagnostic or prognostic marker and therapeutic target for clinical applications.

Other studies have suggested its potential as a diagnostic biomarker and therapeutic target for CC, wherein it functions as an oncogene. Wang et al.^43^ revealed that METTL3 is significantly upregulated and facilitates the proliferation and aerobic glycolysis of CC cells, indicating its intimate association with metastasis and an unfavourable prognosis. Further investigations demonstrated that METTL3 recruits YTHDF1 to target the 3′‐untranslated region of hexokinase 2 (*HK2*) mRNA, thereby enhancing *HK2* stability to exert its function.^43^ Taken together, these findings suggest the potential of METTL3 as a novel factor in triggering the occurrence and development of CC by targeting *HK2* mRNA, thereby suggesting its therapeutic potential for CC treatment.^43^ Another study demonstrated that the heightened METTL3 expression enhances *RAB2B* mRNA stability (a member of the *RAS* oncogene family) in an insulin like growth factor 2 mRNA binding protein 3 (IGF2BP3)‐dependent manner,^44^ ultimately promoting CC cell proliferation and closely correlating with a bleak prognosis in patients with CC.^44^ Collectively, these findings suggest that METTL3 stimulates CC cell proliferation by increasing *RAB2B* mRNA expression, implying that METTL3 may serve as a promising target for CC therapy.

**FIGURE 3:**
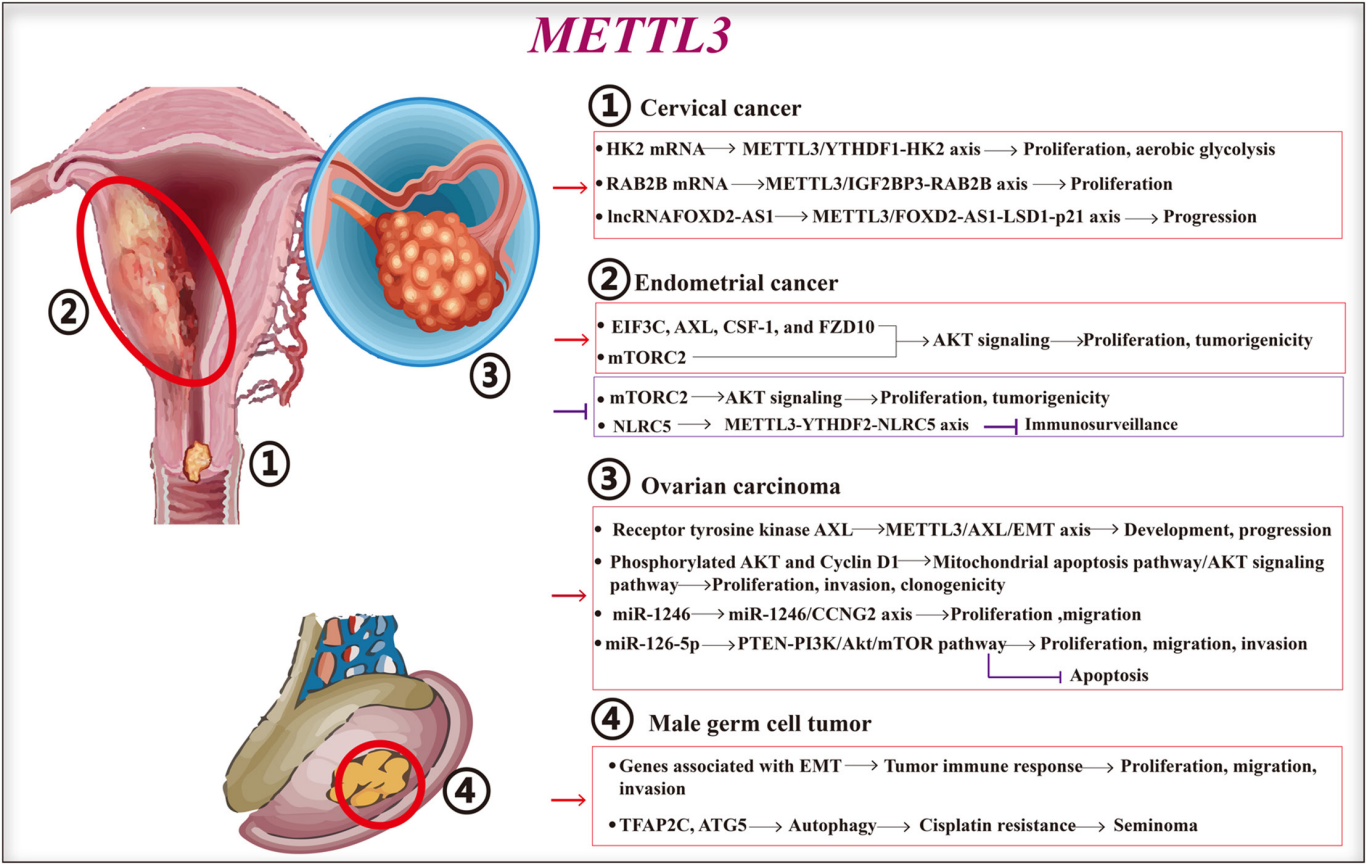
Functions and mechanisms of METTL3 in reproductive tumours. METTL3 is abnormally overexpressed in cervical cancer, endometrial cancer, ovarian carcinoma and germ cell tumour. Overexpression of METTL3 promotes tumour occurrence and progression by targeting RNAs via different mechanisms. The targets, mechanisms and functions of high levels of METTL3 in these tumours are different. This figure classified and comprehensively summarized the targets, pathways and functions of METTL3 in these tumours. ATG5, autophagy related 5; AXL, AXL receptor tyrosine kinase; CCNG2, cyclin G2; CSF1, colony stimulating factor 1; EMT, epithelial‐mesenchymal transition; FOXD2‐AS1, FOXD2 adjacent opposite strand RNA 1; FZD10, frizzled class receptor 10; HK2, Hexokinase 2; IGF2BP3, insulin like growth factor 2 mRNA binding protein 3; METTL3, methyltransferase‐like 3; PTEN, phosphatase and tensin homologue; RAB2B, a member of the RAS oncogene family; TFAP2C, transcription factor activating enhancer‐binding protein 2C.

In addition to targeting mRNA, METTL3 modulates noncoding RNAs to orchestrate CC progression. Ji et al.^45^ revealed that METTL3 amplifies the stability of long noncoding RNA (lncRNA) FOXD2 adjacent to opposite strand RNA 1 (*FOXD2‐AS1*) and sustains its expression, which subsequently recruits lysine‐specific demethylase 1 (LSD1) to the promoter region of p21 to suppress its transcription, ultimately promoting the migration and proliferation of CC cells. In conclusion, these findings suggest that the METTL3/FOXD2‐AS1‐LSD1‐p21 axis promotes CC progression through an m^6^A‐dependent mechanism, thereby presenting a promising therapeutic target for this disease (Figure 3).

In terms of the therapeutic potential of METTL3 in CC, Xu et al.^46^ demonstrated that quercetin enhanced the antitumor effects of cisplatin by inhibiting proliferation, migration, and invasion, while promoting apoptosis in cancer cells by downregulating matrix metalloproteinase 2, EZRIN, METTL3 and P‐GP expression. Despite the significant role of METTL3 as an oncogene that triggers and exacerbates CC, several studies have revealed its anti‐oncogenic role. For instance, Huang et al.^47^ reported that METTL3 regulates miR‐193b maturation through m^6^A‐dependent mechanisms, which act as tumour suppressors by inhibiting CC tumorigenesis and targeting cyclin D1 (*CCND1*). Moreover, Li et al.^48^ reported that METTL3 exhibits higher expression levels in paracancerous tissues than in CC tissues. Functionally, upregulated levels of METTL3 inhibit cell viability and increase apoptosis while enhancing cisplatin sensitivity in CC cells.^48^ Mechanistically, high levels of METTL3 function by downregulating the expression of receptors for advanced glycation end products.^48^

To summarize, despite contrasting findings regarding the expression and function of METTL3, most studies have consistently reported elevated levels of METTL3 in CC, highlighting its oncogenic role in initiating and exacerbating the occurrence and progression of CC. Therefore, it is reasonable to consider elevated METTL3 levels as a promising biomarker for diagnosis and prognosis as well as a potential therapeutic target for future CC therapies.

### 4.2 METTL3 functions in EC need further exploration

EC, originating in the uterus, has been found to exhibit a reduction in m^6^A methylation of approximately 70%, which is likely attribute to the decreased expression of METTL3.^49^ These alterations result in the increased proliferation and tumorigenicity of EC cells by activating the AKT pathway. Diminished m^6^A methylation leads to a reduced expression of the negative AKT regulator PH domain and leucine rich repeat protein phosphatase 2 and elevated expression of the positive AKT regulator mTORC2.^49^ Collectively, these findings reveal that m^6^A mRNA methylation is an oncogenic mechanism in EC and identify m^6^A methylation as a modulator of AKT signalling.^49^ Besides, a cohort analysis of the EC tissue microarray found that the suppressed expression of METTL3 is strongly linked to a poor prognosis in EC patients, which functioned to hinder immunosurveillance by mediating m^6^A modification on *NLRC5* mRNA and promoted NLRC5 degradation by the recognition of YTHDF2.^50^ These findings suggest that the interaction between METTL3 and YTHDF2 decided the fate of *NLRC5* mRNA, which might be a promising target for EC immunotherapy. However, the comprehensive immunohistochemical analysis conducted by Ralser et al.^17^ indicated that METTL3 is abnormally overexpressed and the high level of METTL3 expression predicates poor OS in EC.

Furthermore, another study reported that the expression of METTL3 is elevated in tissues affected by endometrial epithelial OVC.^31^ Functionally, this regulation promotes cell proliferation and migration while simultaneously inhibiting apoptosis in OVC.^51^ Mechanistically, METTL3 functions by elevating m^6^A enrichment within genes linked to OVC, such as eukaryotic translation initiation factor 3 subunit C, AXL receptor tyrosine kinase (*AXL*), colony stimulating factor 1 and frizzled class receptor 10.^51^ Increased METTL3 expression has been used as an indicator of malignancy and a poor prognosis in patients with EC.^51^ Therefore, downregulation of METTL3 may hold potential as a therapeutic approach (Figure 3).

To summarize, the studies on METTL3 and EC have reported contrasting conclusions regarding METTL3 expression and function. Therefore, the relationship between METTL3 and EC remains unclear.

### 4.3 METTL3 triggers and worsens OVC progression via its oncogenic role

Ovary is primarily composed of three distinct cell types, each of which has the potential to develop into a variety of tumours, including epithelial, germ and stromal.^52^ Of these, epithelial cell tumours are the most common.^52^ Recent studies have revealed that METTL3 upregulation in OVC is closely linked to tumour grade and OS rate.^53^ This increase in cellular proliferation promotes focal formation, motility and invasion in OVC cell lines.^53^ Mechanistically, the elevated expression of METTL3 exerts a pivotal oncogenic function in OVC development and progression by promoting epithelial‐mesenchymal transition (EMT) via upregulation of AXL.^53^ These findings collectively suggest that METTL3 can be regarded as a novel prognostic and therapeutic target for OVC, given its ability to stimulate AXL translation toward EMT.

Liang et al.^54^ revealed a marked elevation in METTL3 expression in OVC tissues, which correlated with the presence of large tumours, lymph node metastasis and an elevated pathological grade. Increased levels of METTL3 facilitated cell proliferation, invasion and clonogenicity while suppressing apoptotic rates in OVC cells.^54^ Mechanistically, METTL3 activates the mitochondrial apoptotic pathway and increases the expression of phosphorylated AKT and CCD1.^54^ Collectively, these findings suggest that METTL3 acts as an oncogene that drives human OVC cell progression through the AKT signalling pathway, underscoring its potential as a therapeutic target for OVC. Another study revealed hypomethylation and elevated METTL3 expression in OVC tissues and cells.^55^ Hypomethylation of METTL3 correlates with poor patient survival, whereas elevated levels of METTL3 expression facilitate the proliferation and migration of OVC cells while inhibiting apoptosis.^55^ Mechanistically, METTL3 exacerbates OVC by targeting miR‐1246, which subsequently targets and suppresses cyclin G2 (CCNG2) expression.^55^ These findings collectively underscore the significant role of METTL3 in OVC development through the miR‐1246–CCNG2 axis, indicating its oncogenic potential and identifying novel therapeutic targets.

Furthermore, elevated levels of METTL3 in OVC facilitate the maturation of miR‐126‐5p through the m^6^A modification of pri‐miR‐126‐5p.^56^ Consequently, augmented expression of miR‐126‐5p stimulates proliferation, migration, and invasion, while suppressing apoptosis in OVC cells.^56^ Mechanistically, mature miR‐126‐5p binds directly to phosphatase and tensin homologue, thereby activating the PI3K/AKT/mTOR pathway.^56^ In conclusion, these findings suggest novel tumorigenic mechanisms of m^6^A modification mediated by METTL3 and underscore the potential of targeting METTL3 and miR‐126‐5p for future OVC treatment (Figure 3).

To summarize, significantly elevated levels of METTL3 function as an oncogenic gene, triggering the occurrence and progression of OVC, participating in the promotion of cell proliferation, migration, and invasion, while inhibiting tumour cell death, such as apoptosis. Therefore, it is plausible and rational to use elevated METTL3 expression as a biomarker for OVC diagnosis and prognosis, and as a potential therapeutic target.

### 4.4 METTL3: A marker for poor prognosis in GCT and a therapeutic target for overcoming cisplatin resistance in seminoma

Male GCTs are the most prevalent malignancies affecting male patients aged 15–35 years.^57^ A previous study revealed that METTL3 expression is significantly downregulated in GCT tissues, and individuals with low levels of METTL3 expression exhibit lower OS and DFS rates.^58^

Furthermore, the expression level of METTL3 positively correlates with that of molecular markers and the infiltration levels of CD8^+^ and CD4^+^ T cells and natural killer cells.^58^ Further investigation revealed that METTL3 plays a key role in promoting the proliferation, migration and invasion of GCT cells by modulating the expression of EMT‐associated genes.^58^ Additionally, it may play a pivotal role in activating the tumour immune response in GCT.^58^ In summary, these findings suggest that low METTL3 levels may serve as an independent prognostic marker in patients with GCT.

Chen et al.^59^ reported that an elevated level of METTL3 could potentiate resistance to cisplatin through the m^6^A modification of transcription factor‐activating enhancer‐binding protein 2C in seminoma, indicating the oncogenic role of high levels of METTL3. They discovered a marked upregulation of METTL3 in a cisplatin‐resistant TCam‐2 cell line derived from seminoma, which led to an increase in autophagy and a decrease in sensitivity to cisplatin.^59^ Mechanistically, METTL3 exerts its function by augmenting m^6^A modification levels in the autophagy related 5 (*ATG5*) transcript, thereby enhancing ATG5 expression as a potential target for the METTL3‐mediated promotion of autophagy.^59^ In summary, these findings revealed that METTL3‐mediated m^6^A methylation through *ATG5* targeting exerts regulatory control over autophagy and chemotaxis in TCam‐2 cells. This highlights the potential of METTL3 as a therapeutic target to overcome cisplatin resistance in seminoma (Figure 3).

To summarize, METTL3 expression is significantly upregulated in GCT and downregulated in seminomas, suggesting a distinct role for METTL3 in male reproductive tumours. Low expression of METTL3 as a prognostic biomarker is associated with lower OS and DFS rates in GCT and exerts therapeutic effects in seminomas. Therefore, caution should be exercised when applying METTL3 clinically for male tumours.

## 5 DISCUSSION AND PERSPECTIVES

The m^6^A modification of RNAs primarily relies on the function of METTL3, which affects various biological processes, including its widespread involvement in the reproductive system.^5^ In this review, studies exploring the roles and mechanisms of METTL3 in the reproductive system are comprehensively summarized and interpreted with the aim of elucidating the intricate relationships and potential applications of METTL3 in the reproductive system.

This review shows that an increased level of METTL3 is crucial for spermatogenesis, follicle development, and gametogenesis, which are primarily responsible for promoting the differentiation and maturation of spermatogonia, oocytes, and gametes. Conversely, elevated levels of METTL3 predominantly facilitate cancer occurrence and progression in the female reproductive system. For instance, regarding the function of METTL3 in CC, while an initial study has suggested that high levels of METTL3 suppress tumorigenesis during the cisplatin treatment of CC, the majority of subsequent studies have demonstrated its oncogenic role by targeting mRNA or lncRNAs to promote proliferation and aerobic glycolysis in CC cells. This triggers the progression of CC. These disparate results may be attributed to the distinct samples detected and the diverse mechanisms exerted by METTL3. Hence, it is reasonable to regard high levels of METTL3 as a biomarker for diagnosis and prognosis and as a therapeutic target for future CC treatment. Additionally, an ambiguous expression pattern of METTL3 has been observed in EC, with its levels being either low or high during disease onset and progression. This could be attributed to its dual role as an oncogene and a regulator of other oncogenes. However, given that there are currently limited studies on the association of METTL3 with EC, it is premature to draw any definitive conclusions about their relationship. Hence, extensive investigations that focus on how METTL3 affects EC, encompassing its expression in primary and treated patients, functions, and mechanisms, should be conducted in the future. Furthermore, the upregulation of METTL3 plays a pivotal role in both the onset and progression of OVC. This is because of its ability to not only promote cell proliferation, migration, and invasion, but also to inhibit tumour cell apoptosis. As such, high levels of METTL3 expression can serve as an invaluable biomarker for both the diagnosis and prognosis of OVC, while simultaneously presenting itself as a promising therapeutic target.

In addition, this review clarifies certain regulatory functions of METTL3 in male reproductive disorders. Notably, increased expression of METTL3 is a risk factor for asthenozoospermia because it reduces sperm motility, and subsequently triggers the onset and progression of this condition. Therefore, METTL3 could serve as a promising diagnostic biomarker and therapeutic target for asthenozoospermia in a clinical setting. Interestingly, in GCT, a low expression level of METTL3 serves as an unfavourable prognostic marker, promoting the proliferation, migration and invasion of GCT cells by regulating tumour EMT processes and immune responses. Conversely, in seminomas, high levels of METTL3 play an oncogenic role by enhancing cisplatin resistance during treatment, indicating that lower levels of METTL3 may be beneficial in seminoma therapy. In summary, METTL3 overexpression is deleterious to asthenozoospermia and seminoma chemotherapy, but is therapeutic for GSC.

Based on the information obtained from recent studies, this review suggests that METTL3 functions not only essential for normal development, but also detrimental in the occurrence of disorders in the reproductive system. In addition, the review highlights the potential of using METTL3 for the diagnosis, prognosis, and treatment of some reproductive diseases, such as CC, OVC and GCT.

Although there have been relatively extensive studies on METTL3 in relation to reproductive events, current investigations regarding the ability to fully elucidate the relationship and potential applications of METTL3 in the reproductive system are still lacking. In addition, the full extent of METTL3 implication in reproductive diseases, such as EC, infertility, and other disorders, remains largely unknown, and its function and mechanisms are yet to be fully elucidated. In contrast, despite numerous studies demonstrating the therapeutic potential of targeting either METTL3 or its associated signalling pathways for reproductive diseases, there is still a scarcity of credible clinical data supporting this approach. Therefore, further investigation of the roles and mechanisms of action of METTL3 in the reproductive system is required. Additionally, clinical studies elucidating the potential applications of METTL3 in the prognosis of different tumour stages should be conducted.

Collectively, this review has largely elucidated the relationship between METTL3 and the reproductive system and provides novel insights, potential applications, and future perspectives on the role of METTL3 in regulating the reproductive system, which may be a valuable reference for researchers and clinicians.

## AUTHOR CONTRIBUTIONS

**Xiaojuan Su:** Conceptualization (lead); writing – original draft (lead). **Ruifeng Lu:** Supervision (equal); validation (equal). **Yi Qu:** Conceptualization (equal); writing – review and editing (equal). **Dezhi Mu:** Funding acquisition (lead); supervision (lead).

## FUNDING INFORMATION

This work was supported by the National Natural Science Foundation of China (grant numbers 81971433, 82271749, 82371717 and 81971428).

## CONFLICT OF INTEREST STATEMENT

The authors declare that they have no competing interests.
